# The ambulatory transoral endoscopic thyroidectomy vestibular approach is safe and economical for patients with thyroid nodules

**DOI:** 10.3389/fendo.2023.1116280

**Published:** 2023-02-10

**Authors:** Haiqing Sun, Yongli Chu, Guojun Zhang, Guibin Zheng, Haitao Zheng

**Affiliations:** ^1^ Department of Thyroid Surgery, The Affiliated Yantai Yuhuangding Hospital of Qingdao University, Yantai, Shandong, China; ^2^ Office of Academic Research, The Affiliated Yantai Yuhuangding Hospital of Qingdao University, Yantai, Shandong, China; ^3^ Department of Thyroid and Breast Surgery, Changle People’s Hospital Affiliated to Weifang Medical College, Weifang, Shandong, China

**Keywords:** transoral endoscopic thyroidectomy vestibular approach, ambulatory, safety, mental health, hospitalization cost

## Abstract

**Background:**

Ambulatory thyroid surgery has been increasingly performed in recent years. However, the feasibility of the ambulatory transoral endoscopic thyroidectomy vestibular approach (TOETVA) has not been evaluated. We aimed to evaluate the safety, economy, and mental health outcomes of ambulatory TOETVA.

**Methods:**

We retrospectively reviewed the data of patients who underwent TOETVA between March 2019 and August 2022. The procedure was performed by a skilled surgical team from the Department of Thyroid Surgery of the affiliated Yantai Yuhuangding Hospital of Qingdao University. Patients were enrolled in the ambulatory (n=166) and conventional (n=290) groups, based on their chosen procedure. We analyzed patients’ clinical characteristics, surgical outcomes, Hamilton Anxiety Rating Scale (HAM-A) scores, and hospitalization costs.

**Results:**

Of 456 patients, 166 underwent ambulatory TOETVA and 290 underwent conventional TOETVA. No significant differences were found in clinical and surgical characteristics between the groups, including sex (P=0.363), age (P=0.077), body mass index (P=0.351), presence of internal diseases (P=0.613), presence of Hashimoto’s thyroiditis (P=0.429), pathology (P=0.362), maximum tumor diameter (P=0.520), scope of surgery (P=0.850), or operative time (P=0.351). There were no significant differences in maximum tumor diameter (P=0.349), extrathyroidal tissue invasion (P=0.516), number of retrieved central lymph nodes (P=0.069), or metastatic central lymph nodes (P=0.897) between the groups. No significant differences were found in complications, including transient hypoparathyroidism (P=0.438), transient vocal cord palsy (P=0.876), transient mental nerve injury (P=0.749), permanent mental nerve injury (P=0.926), and other complications (P=1.000). Ambulatory patients had shorter hospital stays (P<0.001) and reduced hospitalization costs (P<0.001). There was no significant difference in HAM-A scores between the groups (P=0.056).

**Conclusions:**

Ambulatory TOETVA is a safe, feasible, and cost-effective procedure for selected patients. This procedure resulted in shorter hospital stays, decreased medical costs, and did not increase patient anxiety. To ensure patient safety, surgical teams must inform patients of the indications, when to seek help, and how to receive the fastest medical attention.

## 1 Introduction

Transoral endoscopic thyroidectomy vestibular approach (TOETVA) has become one of the most common remote access techniques to avoid visible scarring of the neck since it was first performed by Nakajo et al. and Wang et al. in 2013 (1, 2). Increasing evidence has shown that TOETVA is comparable to open thyroidectomy in terms of safety and efficacy (3, 4), making it acceptable in an increasing number of medical centers in recent years.

Steckler first introduced ambulatory thyroid surgery in 1986 (5). In recent years, improvements in surgical techniques have decreased the incidence of complications in thyroid surgery (6), and the use of ambulatory thyroid surgery has gradually increased. Many international reports have shown the safety and economic benefits of ambulatory thyroid surgery (7, 8). The American Thyroid Association released a consensus statement regarding outpatient thyroid surgery in 2013 (9); however, to the best of our knowledge, there are no such reports for ambulatory TOETVA. Therefore, in this study, we aimed to evaluate the safety, economy, and mental health impact of ambulatory TOETVA.

## 2 Materials and methods

### 2.1 Study population

This retrospective study was approved by the Ethics Committee of the Affiliated Yantai Yuhuangding Hospital of Qingdao University. All patients who underwent TOETVA between March 2019 and August 2022 by a single skilled surgical team at the Thyroid Surgery Department of the affiliated Yantai Yuhuangding Hospital of Qingdao University were included in this study. The indications for TOETVA in our department are as follows: (1) patients who were diagnosed with papillary thyroid carcinoma (PTC) with a maximum tumor diameter of ≤1.5 cm in the upper pole or ≤3 cm in other parts of the thyroid and (2) patients diagnosed with benign tumors with a maximum tumor diameter of ≤6 cm. Patients with the following characteristics were excluded: (1) history of neck surgery or neck radiation therapy, (2) lateral cervical or superior mediastinal lymph node metastasis, (3) tumor invasion into extrathyroidal organs. (4) American Society of Anaesthesiology grade of III or higher, (5) inability to receive prompt medical care after discharge for various reasons such as distance from medical facilities, transportation, and education level.

A total of 456 patients underwent TOETVA in our department from March 2019 to August 2022; 166 received ambulatory TOETVA and 290 received conventional TOETVA. Patients were allowed to freely choose between the two procedures, and multimedia materials were provided to patients on multiple occasions to ensure they knew what to watch for, when to seek help, and how to get the fastest medical attention after discharge.

### 2.2 Preoperative examination

Thyroid ultrasonography, neck computed tomography, and fine needle aspiration cytology were performed to screen patients for indications and rule out contraindications. ​Preoperative laryngoscopy was performed to assess vocal cord function in patients with tumors in the posterior part of the gland or those with hoarseness. Preoperative examinations of the patients were performed within 7 days before admission in the ambulatory group and 1 to 2 days after admission in the conventional group. The patients in the ambulatory group underwent surgery on the day of admission, whereas the patients in the conventional group underwent surgery after completing all the preoperative examinations, which took approximately 2 days. The surgeon and anesthesiologist jointly evaluated the patients before surgery to rule out contraindications to surgery or anesthesia.

### 2.3 Surgical procedures and postoperative management

Treatments on the day of surgery were the same in both groups. All patients were required to abstain from food for at least 6 h and water for at least 2 h before surgery. All patients received intravenous cefuroxime sodium and metronidazole 30 min before anesthesia, which was repeated 6 h and 14 h after surgery. All patients were required to use a chlorhexidine gargle before surgery and continued this until 7 days postoperatively. In case of a total thyroidectomy, 600 mg calcium carbonate and 0.25 µg calcitriol would be administered orally twice a day from 2 days before surgery until the day of surgery.

Patients with benign lesions underwent unilateral or total thyroidectomy according to the size and location of the tumor. We can perform TOETVA if the tumor is no larger than 6 cm in size, while surgery is difficult if the tumor is larger than 4 cm in size. So we need to build a larger workspace to increase the mobility of the tumor, and we need to cut the specimen into long strips in the endo-bag before extracting it. On the other hand, patients diagnosed with PTC *via* fine needle aspiration cytology before surgery or intraoperative frozen pathological examination received unilateral or total thyroidectomy and central lymph node dissection.

The surgical technique was the same in both groups as previously described (10–12). All patients in the two groups underwent TOETVA successfully without conversion to open surgery. Venous blood was collected on the morning of the first postoperative day to measure parathyroid hormone (PTH) levels in the patients who underwent total thyroidectomy. Calcium carbonate 600 mg every 6 h and calcitriol 0.25 µg every 12 h were administered orally if serum PTH levels were lower than 15 pg/ml after surgery. Intravenous calcium supplementation was administered if the patient developed hypocalcemia-related symptoms or if serum calcium levels were lower than 1.8 mmol/L. Postoperative laryngoscopy was performed if the doctors or patients sensed abnormalities in sound. Patients in the ambulatory group were discharged from the ward within 24 h after admission, whereas those in the conventional group on the second postoperative day or later.

### 2.4 Clinical parameters

The Hamilton Anxiety Rating Scale (HAM-A) was used to assess patients’ mental health on discharge day. We reviewed patient medical records to collect data, including sex, age, body mass index (BMI), presence of internal diseases, presence of Hashimoto’s thyroiditis, pathology, maximum tumor diameter, the scope of surgery, operative time, number of retrieved and metastatic central lymph nodes (for patients diagnosed with PTC), extrathyroidal tissue invasion (for patients diagnosed with PTC), complications, HAM-A score, and hospitalization costs.

### 2.5 Statistical analyses

Data were analyzed by independent-samples t-tests, Χ^2^ tests, or Fisher’s exact tests using SPSS software (version 26.0; IBM Corp, Armonk, NY, USA). Differences were considered statistically significant at P-values of <0.05.

## 3 Results

### 3.1 Clinical and surgical characteristics

During the study period, 456 patients underwent TOETVA, of whom 166 received ambulatory TOETVA and 290 received conventional TOETVA. No significant differences were observed in the clinical and surgical characteristics between the two groups (**Table 1**). The percentage of women was 84.9% in the ambulatory group and 87.9% in the conventional group (P = 0.363). The mean age was 33.18 ± 8.13 and 34.58 ± 8.06 years in the ambulatory and conventional groups (P = 0.077), respectively. BMI was 24.46 ± 5.16 kg/m^2^ in the ambulatory group and 24.89 ± 4.55 kg/m^2^ in the conventional group (P = 0.351). Four patients in the ambulatory group and five in the conventional group had hypertension, and one of the five hypertensive patients in the conventional group also had coronary heart disease. Coincidentally, no patient in either group had diabetes. Overall, there were no significant differences between the two groups regarding comorbidities (P = 0.613). A total of 49 patients (29.5%) in the ambulatory group and 96 (33.1%) in the conventional group had Hashimoto’s thyroiditis (P = 0.429). A total of 160 patients (96.4%) in the ambulatory group and 274 (94.5%) in the conventional group were diagnosed with PTC (P = 0.362). The maximum tumor diameter was 0.78 ± 0.48 cm in the ambulatory group and 0.75 ± 0.42 cm in the conventional group (P = 0.520). Fourteen patients (8.4%) in the ambulatory group and 23 (7.9%) in the conventional group underwent total thyroidectomy (P = 0.850). Operative time was 160.93 ± 35.82 min and 166.00 ± 38.18 min in the ambulatory and conventional groups, respectively (P = 0.351).

**Table 1:** Clinical and surgical characteristics.

Variables	Ambulatory group n = 166	Conventional group n = 290	P-value
Sex			0.363
Male	25 (15.1%)	35 (12.1%)	
Female	141 (84.9%)	255 (87.9%)	
Age (years)	33.18 ± 8.13	34.58 ± 8.06	0.077
BMI (kg/m^2^)	24.46 ± 5.16	24.89 ± 4.55	0.351
Internal diseases			0.613
Hypertension	4(2.4%)	5(1.7%)	
Coronary heart disease	0(0%)	1(0.3%)	
Diabetes	0(0%)	0(0%)	
Hashimoto’s thyroiditis			0.429
Yes	49 (29.5%)	96 (33.1%)	
No	117 (70.5%)	194 (66.9%)	
Pathology			0.362
PTC	160 (96.4%)	274 (94.5%)	
Benign	6 (3.6%)	16 (5.5%)	
Maximum tumor diameter (cm)	0.78 ± 0.48	0.75 ± 0.42	0.520
Extend of surgery			0.850
Hemithyroidectomy	152 (91.6%)	267 (92.1%)	
Total thyroidectomy	14 (8.4%)	23 (7.9%)	
Operation time (min)	160.93 ± 35.82	166.00 ± 38.18	0.164

BMI, body mass index; PTC, papillary thyroid carcinoma.

A subgroup analysis of the pathological results was performed for patients with PTC in both groups to ensure a similar surgical difficulty between the two groups (**Table 2**). There were no significant differences between the PTC subgroups in maximum tumor diameter (ambulatory, 0.76 ± 0.47 cm vs. conventional, 0.72 ± 0.38 cm, P = 0.349), extrathyroidal tissue invasion (P = 0.516), number of retrieved central lymph nodes (ambulatory, 7.73 ± 4.47 vs. conventional, 6.97 ± 3.98, P = 0.069), and number of metastatic central lymph nodes (ambulatory, 1.41 ± 2.35 vs. conventional, 1.43 ± 2.07, P = 0.897).

**Table 2:** Pathological results of patients diagnosed with papillary thyroid carcinoma.

Variables	Ambulatory group n = 160	Conventional group n = 274	P-value
Maximum tumor diameter (cm)	0.76 ± 0.47	0.72 ± 0.38	0.349
Extrathyroidal tissue Invasion			0.516
Yes	16 (10%)	33 (12%)	
No	144 (90%)	241 (88%)	
Central lymph nodes			
Retrieved	7.73 ± 4.47	6.97 ± 3.98	0.069
Metastatic	1.41 ± 2.35	1.43 ± 2.07	0.897

### 3.2 Complications

Postoperative complications are shown in **Table 3**. Five patients (3.0%) in the ambulatory group and 13 (4.5%) in the conventional group experienced transient hypoparathyroidism (P = 0.438). All the 18 patients who developed transient hypoparathyroidism underwent total thyroidectomy and central neck dissection, and the incidence of hypoparathyroidism in patients who underwent total thyroidectomy and central neck dissection was 48.6% (18/37). Only 2 of the 18 patients with temporary hypoparathyroidism had hypocalcemia-related symptoms on the day of surgery, and their symptoms were rapidly relieved after receiving one dose of intravenous calcium. None of the patients had serum calcium levels of <1.8 mmol/L. Four patients (2.4%) in the ambulatory group and five (1.7%) in the conventional group developed transient vocal cord palsy (P = 0.876). All patients with transient hypoparathyroidism or vocal cord palsy recovered within 3 months. No patients in either group had permanent hypoparathyroidism or vocal cord palsy. Patients who experienced numbness around the jaw area were considered to have sustained mental nerve injury, and those whose condition did not recover in six months after surgery were considered to have permanent mental nerve injury. No significant differences were observed in the percentage of patients with transient (ambulatory, 41.6% vs. conventional, 43.1%, P = 0.749) or permanent (ambulatory, 1.8% vs. conventional, 2.4%, P = 0.926) mental nerve injury between the groups.

**Table 3:** Postoperative complications.

Variables	Ambulatory group n = 166	Conventional group n = 290	P-value
Hypoparathyroidism			
Transient	5 (3.0%)	13 (4.5%)	0.438
Permanent	0 (0%)	0 (0%)	1.000
Vocal cord palsy			
Transient	4 (2.4%)	5 (1.7%)	0.876
Permanent	0 (0%)	0 (0%)	1.000
Mental nerve injury			
Transient	69 (41.6%)	125 (43.1%)	0.749
Permanent	3 (1.8%)	7 (2.4%)	0.926
Other complications			1.000
Seroma	0 (0%)	1 (0.3%)	
Infection	0 (0%)	1 (0.3%)	
Dehiscence of incision	1 (0.6%)	1 (0.3%)	
Tracheal fistula	1 (0.6%)	0 (0%)	
Bleeding	0 (0%)	0 (0%)	
Readmission	0 (0%)	0 (0%)	1.000
PTC recurrence	0 (0%)	1 (0.3%)	1.000

PTC, Papillary thyroid carcinoma.

In the conventional group, one patient developed a seroma and recovered after two ultrasound-guided needle aspiration. One patient who had neck pain and fever was diagnosed with an infection of the surgical site and recovered after intravenous administration of cefoperazone sulbactam for 2 days. One patient who developed partial dehiscence of the intraoral incision recovered within 1 week without re-suturing. In the ambulatory group, intraoral incision dehiscence occurred in one patient on the first day after surgery and healed well without infection after re-suturing. One patient developed subcutaneous emphysema owing to tracheal injury, which healed after intravenous administration of cefoperazone sulbactam and high negative pressure drainage for 13 days. No readmission or postoperative bleeding occurred in either group. The median follow-up was 18 months (range: 3–44 months). Only one patient with PTC in the conventional group experienced recurrence in the contralateral lobe 15 months after the initial surgery. We performed contralateral lobectomy and central neck dissection using a gasless trans-axillary approach, without complications.

### 3.3 Hospital stays, hospitalization cost, and mental health

As shown in **Table 4**, patients in the ambulatory group had shorter hospital stays (1.08 ± 1.01 days vs. 3.41 ± 0.62 days, P < 0.001) and lower hospitalization cost (23,894.47 ± 3298.43 yuan vs. 25,474.30 ± 2686.54 yuan, P < 0.001). The two groups had no significant difference in HAM-A score (7.73 ± 4.20 vs. 6.93 ± 4.33, P = 0.056).

**Table 4:** Hospital stay, hospitalization costs, and impact on mental health.

Variables	Ambulatory group n = 166	Conventional group n = 290	P-value
HAM-A score	7.73 ± 4.20	6.93 ± 4.33	0.056
Hospital stay (days)	1.08 ± 1.01	3.41 ± 0.62	<0.001
Hospitalization cost (yuan)	23894.47 ± 3298.43	25474.30 ± 2686.54	<0.001

HAM-A, Hamilton Anxiety Rating Scale.

## 4 Discussion

The incidence of thyroid disease is high worldwide, and increasing healthcare cost is becoming a concern for many medical centers. Moreover, the COVID-19 pandemic has changed many ways we live and work. Unlike in many European and American countries, we are still implementing strategies to prevent the spread of COVID-19 in China. An ambulatory model can reduce hospital exposure and the chance of infectious disease transmission. Many large-sample studies have confirmed the safety of ambulatory thyroid surgery (13–15). In addition, TOETVA is becoming popular worldwide because of its excellent cosmetic effects. In consensus with our previous study (10, 16), many recent studies have reported that TOETVA is comparable in safety and efficacy to conventional open surgery (3, 4, 17). Therefore, the implementation of ambulatory TOETVA is feasible. This is the first study to evaluate the safety, economic, and mental health impact of ambulatory TOETVA.

In this study, we first analyzed the clinical and surgical characteristics of the patients in the two groups and the pathological results of patients diagnosed with PTC in the two groups. No significant differences were found between the two groups. These results suggest that the surgical difficulty of the two groups was the same, and their complications, hospitalization costs, and mental health impact were directly comparable.

Safety is the biggest concern of ambulatory TOETVA. Akritidou et al. reviewed the complications of the TOETVA (18). Serious postoperative complications, such as asphyxia due to hematoma, bilateral recurrent laryngeal nerve (RLN) palsy, and severe hypocalcemia, may occur after TOETVA. These can be life-threatening if the patient does not receive the necessary medical care after discharge. Minor complications include mental nerve injury, injury of the external branch of the superior laryngeal nerve, seroma, hematoma, subcutaneous emphysema, infection, skin injury, pulling sensation, swallowing disorder, and carbon dioxide-related complications. However, in our study, no patient developed serious postoperative complications

Hematoma is a rare but potentially lethal complication of TOETVA. In our study, none of the patients in the two groups developed hematomas, possibly because we used Hem-o-locks to clamp vessels with a diameter of >3 mm, reducing the risk of postoperative bleeding. Previous studies have also reported a very low incidence of hematoma after TOETVA (10–12, 17, 18). Akritidou et al. reported only 0.53% (10/1887) incidence of hematoma after TOETVA (18), and only 2 of the 10 hematoma patients needed repeat surgery (19, 20). Suzuki et al. (21) analyzed 1,123 patients with neck hematoma after thyroidectomy using the Japanese Diagnosis Procedure Combination database. They concluded that 81.9% (920/1123) of the patients developed a hematoma on days 0 and 1 after thyroidectomy. Carvalho et al. (22) reported that 62.5% (35/62) of patients had neck hematoma within 6 h, and only 12.5% (7/62) more than 24 h after thyroidectomy. Although there are no extensive sample reports, we can infer from the occurrence time of hematoma after open thyroid surgery that most hematomas occur shortly after TOETVA.

RLN injury is a common complication of TOETVA. Nine patients (2.0%) in our study developed transient RLN injury, with no significant difference between the groups; however, none of the patients developed permanent RLN injury in either group. Studies have shown post-TOETVA incidence to be 0.5%–5.9% and 0%–2.3% for transient and permanent RLN injuries, respectively (11, 19, 23, 24). All cases of RLN injury in previous studies were unilateral; to the best of our knowledge, there are no reports of bilateral RLN injury after TOETVA. Unilateral RLN injury, which causes hoarseness only, is not potentially life-threatening and does not require hospitalization; therefore, it does not interfere with ambulatory procedures.

As Akritidou et al. reported, the incidence of transient and permanent hypoparathyroidism in TOETVA was 6.11% (range: 0.94%–22.2%) and 0.21% (range: 1.33%–2.22%), respectively (18). However, lobectomy and nodulectomy accounted for most TOETVA operations in the study by Akritidou et al., while total thyroidectomy accounted for only a small proportion. As we reported previously (10), transient hyperparathyroidism occurred in 67.9% of patients with total oral thyroidectomy and bilateral central lymph node dissection. In the current study, the incidence of hypoparathyroidism in patients with total thyroidectomy and central neck dissection was 48.6% (18/37). Therefore, special attention should be paid to calcium supplementation and serum calcium monitoring during the perioperative period to prevent severe hypocalcemia. Mattoo et al. (25) reported a hypocalcemia rate of 54% in patients who underwent total thyroidectomy. They found that the best cut-off serum PTH value was 4.28 pmol/l (39 pg/ml) 20 min after total thyroidectomy because 23% (17/74) of patients required parenteral calcium supplementation when the serum PTH level was below 4.28 pmol/l and only 5.4% (2/37) of patients required parenteral calcium supplementation when the PTH level was above 4.28 pmol/l. In the current study, the incidence of transient hypoparathyroidism was 3% in the ambulatory group and 4.5% in the conventional group. None of the patients had permanent hypoparathyroidism. Only 5.4% (2/37) of the patients who underwent total thyroidectomy developed transient hypocalcemia-related symptoms. This proportion is lower than that reported by Mattoo et al., possibly because of the prophylactic calcium and calcitriol supplements administered during the perioperative period.

Tracheal injury occurred in one patient in the ambulatory group due to a puncture of the tracheal by an ultrasonic scalpel. A primary suture was immediately performed without conversion to open surgery, and the patient developed a large area of subcutaneous emphysema in the neck after coughing on postoperative day 1. This was the only patient in the ambulatory group with delayed hospital discharge. Tracheal injury is also a rare complication of TOETVA. Long et al. (26) reported two cases of tracheal injury: one was closed after conversion to open surgery, and the other was closed transorally. These two patients were discharged on postoperative days 1 and 2.

Other complications included mental nerve injury, seroma, infection, and dehiscence of the incision. Mental nerve injury is a common complication after TOETVA, with reported incidence of 0.7%–78.57%, most of which were transient (10, 12, 19). Mental nerve injury causes sensorimotor abnormalities of the lower lip and jaw, which do not require a prolonged hospital stay. Seroma, infection, and dehiscence of the incision are all rare complications of TOETVA, with incidence of less than 1% in most studies (10, 12, 16, 19, 24, 27).

While overnight observation can ensure the safety of most patients, there is still the possibility of complications after discharge, including hematoma and severe hypocalcemia. Therefore, the surgical team must strictly educate patients to ensure that they know what to watch for, when to seek help, and how to get the fastest medical attention.

In our study, patients in the ambulatory group had a shorter mean hospital stay and lower mean hospitalization cost. Studies on ambulatory surgery have reported different reduced hospitalization cost (28, 29). Shortening hospital stay improves the efficiency of medical resources, reduces economic burden, and enables more patients to receive surgical treatment.

In our study, there was no significant difference in HAM-A scores between the two groups, indicating that patients in the ambulatory group were no more anxious than those in the conventional group. Zeyu-Zhang reported more anxiety among patients in the ambulatory group (30, 31); this may be because we used multimedia to provide patients with a complete understanding of their condition and how to ensure their safety after discharge, thereby reducing anxiety.

Our study has several limitations: First, as this was a retrospective study, patient selection bias and confounding factors were inevitable. Second, most patients in our study were young and had few medical conditions; therefore, this study cannot be used as evidence for ambulatory TOETVA in patients with medical conditions or elderly patients. Third, the sample size in this study was not large enough to assess the impact of rare complications on safety, especially in patients treated with bilateral thyroidectomy. Fourth, the patients were not evaluated for vocal cord paralysis by routine laryngoscopy after surgery, which can lead to an underestimation of vocal cord paralysis. Finally, costs incurred at other medical centers after discharge were not included because they were not available in our system.

In conclusion, our study evaluated the safety, economic, and mental health impact of ambulatory TOETVA, which had not been reported in previous studies. The results showed that in skilled surgical centers, ambulatory TOETVA is a safe, feasible, and cost-effective procedure for selected patients. The reduction in hospital stay associated with TOETVA did not increase patient anxiety. The surgical team must strictly educate patients to ensure they know what to watch for, when to seek help, and how to get the fastest medical attention to ensure patient safety.

## Data availability statement

The original contributions presented in the study are included in the article/**Supplementary Material**. Further inquiries can be directed to the corresponding author.

## Ethics statement

The studies involving human participants were reviewed and approved by Ethical Committee of The Affiliated Yantai Yuhuangding Hospital of Qingdao University. Written informed consent to participate in this study was provided by the participants’ legal guardian/next of kin.

## Author contributions

Study design: HS. Modify the study design: HZ and YC. Data collection: GJZ and HS. Data analysis: HS, YC, and GBZ. Drafting the manuscript: HS. Project supervision: HZ. All authors contributed to the article and approved the submitted version.
